# Sclareol Inhibits Hypoxia-Inducible Factor-1α Accumulation and Induces Apoptosis in Hypoxic Cancer Cells

**DOI:** 10.34172/apb.2022.062

**Published:** 2021-07-04

**Authors:** Somayeh Vandghanooni, Zahra Farajzadeh Vahid, Ailar Nakhlband, Mir Babak Bahadori, Morteza Eskandani

**Affiliations:** ^1^Hematology and Oncology Research Center, Tabriz University of Medical Sciences, Tabriz, Iran.; ^2^Faculty of Natural Sciences, Department of Biology, University of Tabriz, Tabriz, Iran.; ^3^Research Center of Psychiatry and Behavioral Sciences, Tabriz University of Medical Sciences, Tabriz, Iran.; ^4^Medicinal Plants Research Center, Maragheh University of Medical Sciences, Maragheh, Iran.; ^5^Research Center for Pharmaceutical Nanotechnology, Tabriz University of Medical Sciences, Tabriz, Iran.

**Keywords:** Sclareol, Natural compound, A549, Lung cancer, Hypoxia, HIF-1α

## Abstract

***Purpose:*** The hypoxia in solid tumors is associated with the resistance to chemo/radiotherapy. Hypoxia-inducible factor-1 (HIF-1) plays a key role in cell remodeling to hypoxia. Therefore, the inhibition of HIF-1 accumulation is considered a hopeful strategy for the treatment of cancer. Here, we aimed to evaluate the geno- and cytotoxicity properties of sclareol, a natural bicyclic diterpene alcohol, on A549 cells in CoCl_2_-induced hypoxia.

***Methods:*** The cytotoxicity and apoptosis-inducing properties of sclareol on the A549 cell were evaluated using MTT assay and Annexin V/PI staining, respectively in hypoxia. DAPI staining, DNA ladder, and comet assay were used to evaluate the genotoxicity. Further, the qPCR technique was employed to assess the expression of *HIF-1α, HIF-1β,* and downstream target genes (*GluT1,* and *Eno1*). Finally, the level of HIF-1α protein was evaluated through Western blotting in sclareol-treated cells in hypoxia.

***Results:*** The inhibitory concentration (IC_50_) of sclareol against A549 cells was 8 μg/mL at 48 hours in hypoxia. The genotoxicity of sclareol was confirmed in the cells treated with sclareol in hypoxia. Sclareol induced ~46% apoptosis and also necrosis in the hypoxic condition. The qPCR analyses showed an enhanced suppression of HIF-1α, HIF-1β, *GluT1,* and *Eno1* due to the sclareol treatment in the hypoxia. Moreover, protein quantification analysis showed dose-dependently degradation of HIF-1α in hypoxia upon treatment with sclareol.

***Conclusion:*** The results obtained here indicate that sclareol possesses dose-dependent cytotoxicity effects against A549 cells in hypoxia through inhibition of HIF-1α protein accumulation, increasing cell sensitivity to intracellular oxygen levels, and disruption of cell adaptation to hypoxia.

## Introduction

The unlimited proliferation of tumor cells results in a deficiency in nutrition and oxygen.^1^ The low oxygen level in solid tumors stimulates various pathways in cancer cells, leading to cell adaptation to hypoxia, inhibition of cell death, induction of cell proliferation, and enhancement of oxygen delivery into the cells through metabolism changes and neovascularization.^2^ Hypoxia-inducible factor-1 (HIF-1) acts as an oxygen sensor in which is regulated through oxygen-dependent approaches^3^ and also oxygen-independent pathways (e.g., PI3K, AKT, and MAPK).^4^ Various investigations showed that the expression of different proteins involved in metastasis and pre-metastasis of cancer cells is associated with the levels of nucleus HIF-1.^5^ The HIF-1 transcription factor is a family of basic–helix-loop-helix (bHLH) and composed of HIF-1α and HIF-1β subunits.^6^ The HIF-1α level is controlled by the oxygen concentration.^7^ In the normoxia (10-21% oxygen tension), the asparagine 803 in the HIF-1α subunit is hydroxylated leading to the degradation of HIF-1α by the 26S proteasome degradation system. But in hypoxia, it moves to the nucleus, and binds to the HIF-1β, forming a HIF-1 transcription factor.^8^

An autoregulatory feedback mechanism was recommended for HIF-1α degradation in normoxia through HIF-1α proteasome targeting factor (HPTF). It was suggested HPTF causes HIF-1α degradation by the proteasome system through HIF-1-mediated transcriptional activity.^9^ Besides, the HIF-1α gene polymorphisms are associated with the altered expression and enhanced activation of the HIF-1α protein in cancer cells. HIF-1α polymorphisms (1772C > T and 1790G > A) in the N-terminal transactivation domain increase significantly the transcriptional activity of HIF-1α and have been linked to the poor treatment outcomes in various malignancies including lung, breast, oral, prostate, cervical and renal cancers.^10^ The C1772T (P582S) polymorphism of the HIF-1α gene was reported to be correlated with the elevated levels of HIF-1α, lymph node metastasis, and high histological grade in breast cancer.^11^

On the other hand, the HIF-1β is constantly expressed in hypoxia and normoxia, and therefore the information about the HIF-1β subunit and its role in tumor biology is limited. However, upregulation of HIF-1β in response to oxygen deficiency was shown in some tumor cells indicating its possible role in cell proliferation and/or cell survival during tumorigenesis. It was found that HIF-1β is especially required during the early stages of tumor growth.^6^ Also, it was demonstrated that the expression of HIF-1β plays an essential role in cisplatin-resistance cancer cells. The overexpression of HIF-1β upregulates multidrug resistance gene 1 (MDR1) in cancer cells leading to chemoresistance in cancer cells.^12^

HIF-1 induces the expression of downstream target genes involving in cell adaptation to hypoxia.^13^ These genes affect metastasis, metabolisms, angiogenesis, cell proliferation, and survival of hypoxic cancer cells.^14^ HIF-1α and HIF-2α are involved in new blood vessel formation. HIF-1α induces the recruitment of endothelial progenitor cells from the bone marrow and stimulates their differentiation into endothelial cells by the regulation of VEGF. Hypoxia and HIF1-α also are involved in the angiogenesis process by inducing the expression of matrix metalloproteinase (MMP) enzymes to break the pre-existing vessels. Further, hypoxia influences the migration of cancer cells through epithelial-mesenchymal transition (EMT) and alters their gene expression before migration. Reduction in epithelial-associated gene expression (e.g., E-cad and β-catenin) and increasing the mesenchymal-like gene expression (e.g., N-cad and vimentin) are the hallmark of hypoxia-induced EMT in cancer cells.^15^ HIF-1 can initiate hypoxia-induced apoptosis by increasing the expression of Bcl-2 binding proteins and impeding the anti-apoptotic function of Bcl-2. Stabilization of wild-type p53 by HIF-1 is another mechanism for apoptosis induction. However, hypoxia prevents apoptosis through up-regulation of anti-apoptotic protein IAP-2.^16^ All of these changes promote cell growth, malignancy, enhance neovascularization, and inhibit apoptosis. Therefore, the inactivation of HIF-1α is a hopeful approach for the development of new therapeutic modalities.^8,17-19^ Various investigators tried to find anti-cancer lead compounds with inhibitory effects against the accumulation of HIF-1 in the nucleus of hypoxic cells to overcome the hypoxia adaptation and also increase the sensitivity of cells to chemotherapy agents. Our research group recently has focused to find potent compounds with an inhibitory effect against hypoxia adaptation pathway. In this context, we investigate various compounds biological properties with inhibitory effects against the HIF-1 function and found that galbanic acid, clerodermic acid and corosolic acid target HIF-1α and showed potent anti-cancer properties through dysregulation of hypoxia adaptation.^8,19,20^

Sclareol is a natural diterpenoid extracted from clary sage (*Salvia sclarea*).^21^ It is an amber-colored solid with a sugary, balsamic perfume which is known as a flavoring.^22^ It has been found that sclareol has cytotoxic effects in human leukemic cells, colon cancer cells, osteosarcoma, and cervical cancer cells via induction of apoptosis and other molecular mechanisms.^23,24^ We evaluated the cytotoxicity effects of sclareol on A549 in normal oxygen, previously and found that sclareol induces apoptosis on A549 cells in a dose and time-dependent manner.^25^ The effect of sclareol on A549 cells in hypoxia has not been reported, until now. In this study, the effects of sclareol were screened on the A549 cell line in hypoxia, and its inhibitory mechanism was assessed by geno/cytotoxicity assays. Besides, the hypoxia-related genes and protein expression were also evaluated by quantitative real-time PCR (qPCR) and western blot analysis, respectively.

## Materials and Methods

### Chemical and reagents

Sclareol and trypsin (0.25%) were bought from Sigma Aldrich Co. (Poole, UK). A549 human lung epithelial cancer cell line was bought from the national cell bank of Iran, Pasteur Institute (Tehran, Iran). RPMI 1640 medium, fetal bovine serum (FBS), cell culture instruments were acquired from Gibco, Invitrogen (Paisley, UK), and IWAKI (Japan) respectively. The chemicals were provided by Merck Co. (Darmstadt, Germany). Normal melting point agarose (NMP) was purchased from Gibco, Invitrogen (Paisley, UK).

### Cell culture

The RPMI 1640 medium containing FBS (10%), penicillin (100 μg/mL), and streptomycin (100 μg/mL) was employed for the cultivation of A549 cells. The cells were grown in a humidified atmosphere with 5% CO_2_ at 37 °C. For hypoxia (1% oxygen) stimulation, cobalt chloride (CoCl_2_) was used at a concentration of 100 µM.^8,26^ To study the morphological changes of the cells, images were captured by an Olympus DP72 camera (Shinjuku City, Tokyo, Japan) connected to an Olympus IX81 fluorescence microscope (Hamburg, Germany).

### Determination of cell viability

The MTT assay was used to quantify the living/dead cells in the sclareol-treated A549 cells in hypoxia. In this method, yellow 4, 5-dimethylthiazol-2,5-diphenyl tetrazolium bromide (MTT) is reduced in living cells by NADH-dependent oxidoreductase enzymes and tetrazolium is converted to insoluble formazan crystals which can be easily solubilized in DMSO, resulting in the production of purple color which can be measured at 570 nm.^27^ Here, the stock solution of sclareol was firstly sterilized by passing through the 0.2 μm syringe filter. Then, different concentrations of sclareol were prepared with RPMI 1640 medium (DMSO < 0.05% v/v). To avoid the probable cytotoxic effects of DMSO, 0.05% DMSO was utilized as a negative control and the results were normalized against negative control. A549 cells were cultured at a density of 2 × 10^4^ cells/well at 37°C in hypoxia overnight. Then they were incubated with various concentrations of sclareol for 24, 48, and 72 hours. After the incubation time, fresh MTT solution (2 mg*/*mL) was added and the cells were incubated for 4 hours. Then, the MTT solution was removed and DMSO containing Sorensen’s buffer (0.133 M, pH 7.2) was used to dissolve the formazan crystals. The absorbance of each well was measured at 570 nanometers using Biotek ELx800 spectrophotometer (San Francisco, CA, USA). For positive control, DMSO was used at a final concentration of 5%.

### Genotoxicity assays

#### DAPI assay

DAPI staining was done to evaluate the nuclear morphology, and condensed and fragmented DNA in sclareol-treated A549 cells.^28^ First, the wells were covered with the 12 mm coverslips, and then 4.0×10^5^ cells/well were cultured in 6-well plates. The cells were treated with the sclareol at the final concentration of 19 µg/mL (IC_50_ value of sclareol in normal oxygen condition at 48 hours^25^). After 48 hours, paraformaldehyde (4%) was used to fix the cells for 8 minutes. To make the cell membrane permeable, Triton-X-100 (0.1%) was employed for 5 minutes. Then, the cells were stained with DAPI dye (200 ng/mL) and incubated in dark. Eventually, any morphological changes in the nuclei of the normal and apoptotic cells of the sclareol-treated cells in hypoxia were evaluated using the fluorescence microscope.

#### DNA ladder

DNA fragmentation assay was employed to evaluate DNA breakage as an indication of apoptotic or necrotic cells in sclareol-treated cells.^29-31^ After treatment with sclareol, the total DNA of the cells was extracted and then subjected to the agarose gel electrophoresis to visualize the DNA breakage pattern. Briefly, the cells were washed and exposed to the lysis buffer containing 50 mM Tris base, 10 mM EDTA, and 0.5% sodium dodecyl sulfate. Centrifugation was done at 12000×g for 5 minutes and the supernatant was removed. Ice-cold chloroform:isoamyl alcohol (24:1) was used to isolate total proteins and phenols. Next, after centrifugation at 12 000×g for 5 minutes, the DNA in the upper aqueous phase was participated using ice-cold ethanol (70% v/v) and dissolved in DNase/RNase-free water. Finally, the quality and quantity of extracted DNA were assessed by measurement of absorption intensity at wavelengths of 260 and 280 nm. The same amount of DNA samples was loaded on the 2% agarose gel containing SYBR Green I fluorescent dye and imaged by a gel documentation system.

#### Alkaline comet assay

The single-cell gel electrophoresis assay or comet assay is a sensitive method to assess the DNA breakage in cells.^32^ Here, the genotoxicity effects of sclareol on A549 cells in the hypoxic condition were assessed by the comet assay method. According to the previous study,^31^ microscopic slides were covered with NMP agarose (1.5% v/v), and then the cells (1×10^4^ cells) were embedded in the low melting-point (LMP) agarose and placed on the covered slides. After lysis of the cells with Triton X-100 in a dark place, they were placed into an alkaline electrophoresis solution (300 mM NaOH and 1 mM Na_2_EDTA, pH > 13) to unwind DNA strands and remove histone proteins. After then, electrophoresis was done for 30 minutes in cold conditions. After washing slides with neutralization buffer (40 mM Tris–HCl, pH 7.5), ethidium bromide was used to stain the slides and then were immediately visualized with the fluorescence microscope. CASP software was used for the image analysis. The ratio of total fluorescence DNA in the tail to the head was expressed as a single-strand breakage in DNA.

### Annexin V/PI staining for evaluation of apoptosis and necrosis

The extent of apoptosis/necrosis rate in sclareol-treated cells in hypoxia condition was quantified through FITC-labeled annexin V and propidium iodide (PI) staining.^29,30^ In brief, A549 cells were cultured and then treated with sclareol and incubated for 48 h. After harvesting the cells, the annexin V binding buffer was added and then the cells were stained with FITC-labeled annexin V in dark. The cells were then centrifuged for 5 minutes at 300×g and then stained with PI. Finally, the cells were examined using Becton Dickinson BD (FACS) FACSCalibur flow cytometer (San Jose, CA USA). Emission was detected at 515-545 nm and 600 nm for FITC and PI, respectively.

### Cell cycle examination

Sclareol-treated cells were stained with PI (5 μg/mL) to determine cell cycle distribution. Cell cycle analysis evaluates the cells in the sub-G1 phase which indicates the rate of cell death. Briefly, treated cells were detached by the enzyme digestion and then collected through centrifugation at 3000×g for 5 minutes. To fix the cells, the ice-cold ethanol (70%) was applied drop-wise following by incubation at 4°C for 1 hour. The cell pellets were then suspended in the PI staining solution for 30 minutes in the dark which was contained ribonuclease enzyme to avoid RNA staining. Flow cytometry analysis was performed with 10 000 events for each sample. Data evaluation was done with WinMDI 2.8 software.

### Gene expression analysis by qPCR

The quantitative PCR (qPCR) analysis was used to evaluate the hypoxia-related gene expression including *HIF-1α*, *HIF-1β*, and downstream target genes (*GluT1* and *Eno1*) involving in hypoxia adaptation using a Bio-Rad iQ5 real-time PCR system (Hercules, CA, USA). The gene expression in sclareol-treated and untreated cells was quantitatively compared in hypoxia and normoxia. Total RNA was extracted using Qiagen RNA extraction Kit (Hilden, Germany) and the quantity and purity of the isolated RNAs were evaluated by the measurement of absorption at A260 and A280 nm utilizing NanoDrop spectrophotometers (Waltham, MA, USA). Primers were designed using Oligo 7.56 (Molecular biology insights, Inc, USA) as shown in Table 1. Following complementary DNA (cDNA) synthesis,^33,34^ the qPCR was performed in a 25 μL reaction mixture using Applied Biosystems SYBR Green master mixes (Foster City, CA, USA). Data analysis was performed through the Pfaffl method and by normalization of cycle threshold (Ct) values of genes to the GAPDH gene.

**Table 1:** Primer sequences and characteristics

**Genes name**	**Gene Bank accession no**	**Sequences**	**Annealing temperature**	**Amplifying length**
*HIF-1α*	NM_181054.2	HIF-1αF: 5’- CTTACACACAGAAATGGCCTTG-3’	60	133
		HIF-1αR: 5’- ATACCTTCCATGTTGCAGAC-3’		
*HIF-1β*	NM_001197325.1	HIF-1β F: 5’- ACAATCATTCCCAGGTGGTTC-3’	61	148
		HIF-1β R: 5’- GCCTTTACTCTGATCCGCATTG-3’		
*Eno 1*	NM_001428.3	Eno 1 F: 5’- GAGTCTCTTCAGGCGTGCAAG-3’	58	133
		Eno 1 R: 5’- CAGTCTTGATCTGCCCAGTGC -3’		
*GluT-1*	NM_006516.2	GluT1 F: 5’- GTCTGGCATCAACGCTGTCTTC-3’	60	133
		GluT1 R: 5’- CACCACAAACAGCGACACGAC-3’		
*GAPDH*	NM_002046.3	GAPF: 5’-AAGCTCATTTCCTGGTATGACAACG-3’	62	126
		GAPR: 5’-TCTTCCTCTTGTGCTCTTGCTGG-3’		

### Western blot analysis

To perform western blotting analysis, the cells were washed with PBS and lysed with lysis buffer containing 1% Triton X-100, 0.1% SDS, and protease inhibitor cocktail tablets (Roche Applied Science; Penzberg, Germany). The quantity of the extracted proteins was assessed by the nanodrop spectrophotometer at 280 nm.^35^ The equal amount of total protein was then subjected to the polyacrylamide gel electrophoresis (SDS-PAGE). Gels were run under a constant 500 mA current for 45 minutes. TBS (PBS with 0.05% Tween 20) was used to wash the gel.^8^ After transferring the proteins to the nitrocellulose membranes, anti-HIF-1α, and anti-β-actin (as housekeeping protein) were then added to the paper (Invitrogen; Carlsbad, CA, USA) and incubated with secondary antibodies conjugated with horseradish peroxidase (HRP) (Abcam; Cambridge, UK). A chemiluminescence detection kit (Millipore; Burlington, MA, USA) was used to determine the signals from the blotted membrane. The quantity and integrity of the protein bounds were assessed by TotalLab Quant v 12.2 software and the β-actin was used for the normalization of protein expression.

### Statistical analysis

All experiments were repeated three times and obtained data were displayed as mean ± SD. Two groups were compared statistically with Student’s* t*testand multiple comparisons were done through one-way ANOVA analysis. Also, the groups with significant mean differences were compared with Tukey multiple comparisons as a post hocanalysis. The *P* ≤ 0.05 was considered as a significance level.

## Results and Discussion

### Cell morphology changes and cytotoxicity assay

**Figure 1:**
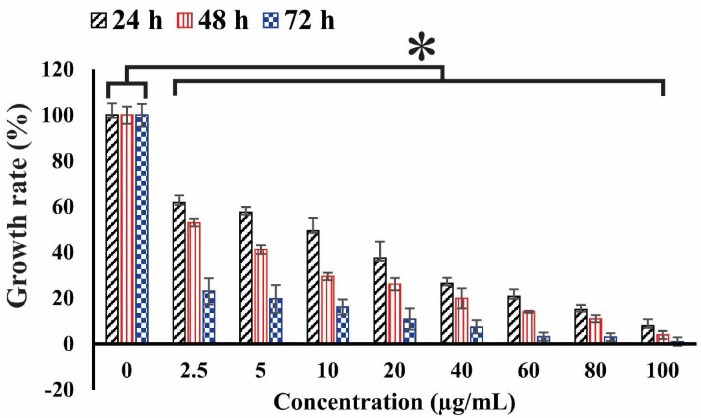
The cell viability of sclareol-treated cells in hypoxia with different concentrations at 24, 48, and 72 h. The cytotoxicity effect of sclareol-treated groups in hypoxia was compared with the untreated negative control. Data represent the mean ± SD of three independent experiments. The asterisk represents significant differences between treatment conditions (*P* ≤ 0.05).

The microscopy images showed significant morphological changes of A549 cells in the presence of sclareol in hypoxia in comparison to the untreated samples (data not shown). The main reason for these differences is the disruption of the actin and myosin protein structure.^36^ Reduction in cell density and an enhancement in the number of floating cells showed the occurrence of cell death in sclareol-treated cells. Previously, we reported the cytotoxicity of plain sclareol and sclareol-loaded SLNs and found that the plain sclareol causes significant toxicity with an IC_50_ of 19 μg/mL against A549 cells in normal oxygen condition.^25^ In this investigation, the MTT assay was employed to determine the cell viability after exposure to sclareol in hypoxia. The cell viability was reduced in the sclareol-treated cells with an increase in dose and treatment time (Figure 1). According to the MTT results, the IC_50_ values were 18, 8, and 4.7 µg/mL under hypoxia in 24, 48, and 72 hours, respectively.

### Genotoxicity assays

**Figure 2:**
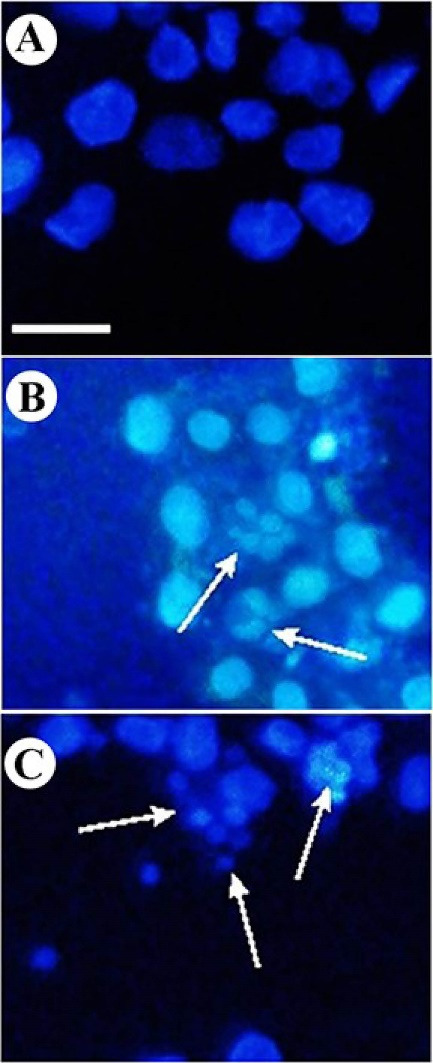
DAPI staining of A549 cells. Change of nuclear morphology and chromatin condensation in (A) untreated cells, (B) sclareol-treated cells in hypoxia, and (C) positive control.

DAPI staining was carried out to assess the sclareol-induced genotoxicity effects on the A549 cells. DAPI produces severe fluorescent light when it binds to the regions with high A-T bases in DNA.^37^ Because of distinct characteristics of the nuclear apoptotic cells, such as chromatin impaction and fragmentation, DAPI staining could be used for the visual identification of the apoptotic cells.^38^ Figure 2 shows the morphology of sclareol-treated cells in hypoxia. DNA fragmentation and chromatin condensation provide intense fluorescence signals, indicating the apoptosis occurrence in treated cells while in the untreated cells (negative control) the morphology remained unchanged. These findings confirmed the genotoxic properties of sclareol on A549 cells in hypoxia.

**Figure 3:**
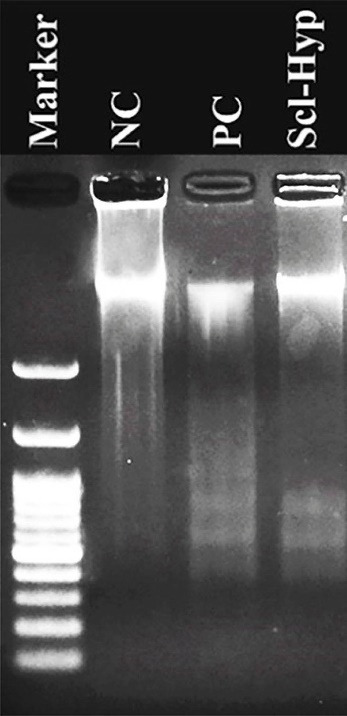
DNA ladder pattern in the NC (negative control), PC (positive control), and sclareol-treated A549 cells in hypoxia (Scl-Hyp).

DNA fragmentation assay was also employed to approve the genotoxic effects of sclareol on A549 cells in hypoxia.^39^ During the occurrence of apoptosis, endonuclease break DNA strands up to 200 bp in large part due to the activation of caspases.^40^ The DNA ladder pattern confirms the occurrence of apoptosis.^41^ As Figure 3shows, the DNA breakage was clear in sclareol-treated cells in hypoxia. The DNA content of cells in the negative control remained intact largely due to a lack of apoptosis.

**Figure 4:**
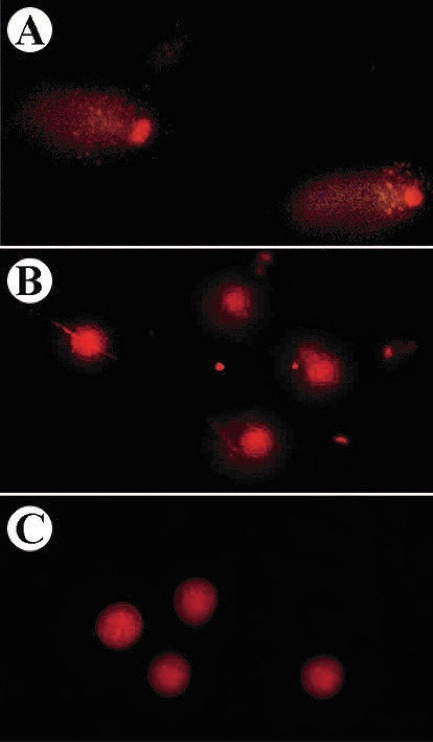
The comet images of sclareol-treated A549 cells in (A) hypoxia, (B) 5% DMSO-treated cells, and (C) untreated A549 cells.

Moreover, an alkaline comet assay was also used to semi-quantitatively evaluate the genotoxicity of sclareol on A549 cells in hypoxia. Comet assay is an accurate technique for evaluating the quantity of damaged DNA and genotoxicity induced by cytotoxic drugs using the microelectrophoresis technique.^42^ Figure 4 shows the microscopic images of comets in the A549 cells. In this method, different microscopic images were taken from different slide areas and then were analyzed by Casp software. After the evaluation of 30 comets, the average of the DNA amount in the tail to DNA amount in the head was calculated and expressed between 0 and 1 which are given in Table 2.

**Table 2:** DNA content in the comet of sclareol-treated cells in hypoxia

**Treatments**	**% Tail DNA/head DNA**
Untreated cells	0.027 ± 0.003**
Sclareol-treated cells-hypoxia	0.298 ± 0.044**
DMSO (5%)-treated cells	0.189 ± 0.048

*Indicated the differences between the treated and untreated cell were significant (P < 0.01).

### Detection of apoptosis/necrosis and cell cycle arrest investigation

**Figure 5:**
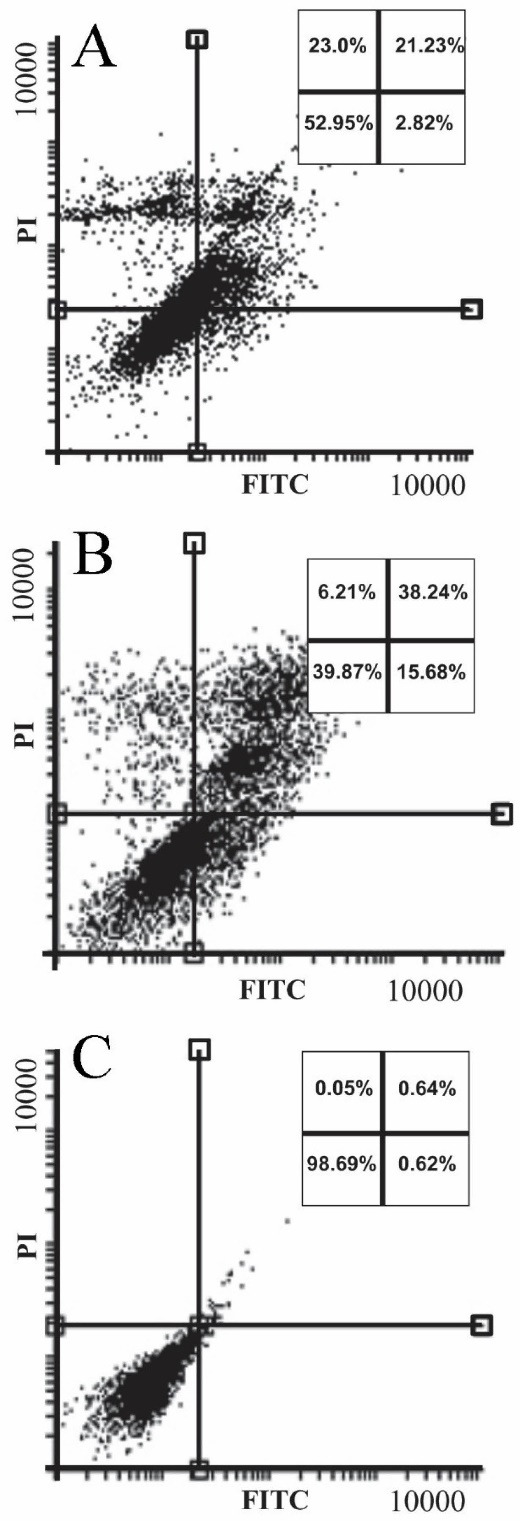
Annexin V/PI staining for detection of apoptosis/necrosis in the (A) sclareol-treated cells. Panel (B) and (C) show the incidence of apoptosis/necrosis in 5% DMSO positive control and untreated cells, respectively.

The apoptotic/necrotic cells in the sclareol-treated cells were determined using Annexin V-FITC/PI-based flow cytometry analysis. In the early apoptotic cells, phosphatidylserines externalize on the plasma membrane which can be detected by FITC-labeled Annexin V. Fluorescent PI binds to the DNA by intercalating between the bases. In the last phase of apoptosis and necrosis, the cell membrane becomes permeable, in which PI enters into cells and stain broken DNA. So, the late apoptotic cells and necrotic cells can be distinguished by PI staining.^43,44^ The apoptosis/necrosis rate of sclareol-treated cells in hypoxia is shown in the apoptosis/necrosis rate of sclareol-treated cells in hypoxia is shown in Figure 5. Data analyses showed that the apoptosis and necrosis rate of the sclareol-treated cells in hypoxia was increased significantly (*P* < 0.05) to 25% and 23%, respectively after 48 hours compared to untreated cells. These findings indicated the activation of apoptosis pathways in sclareol-treated cells in hypoxia. Moreover, as Figure 5shows, the rate of apoptotic and necrotic cells in positive control was 53% and 6% respectively after 48 h treatment with 5% DMSO.

**Figure 6:**
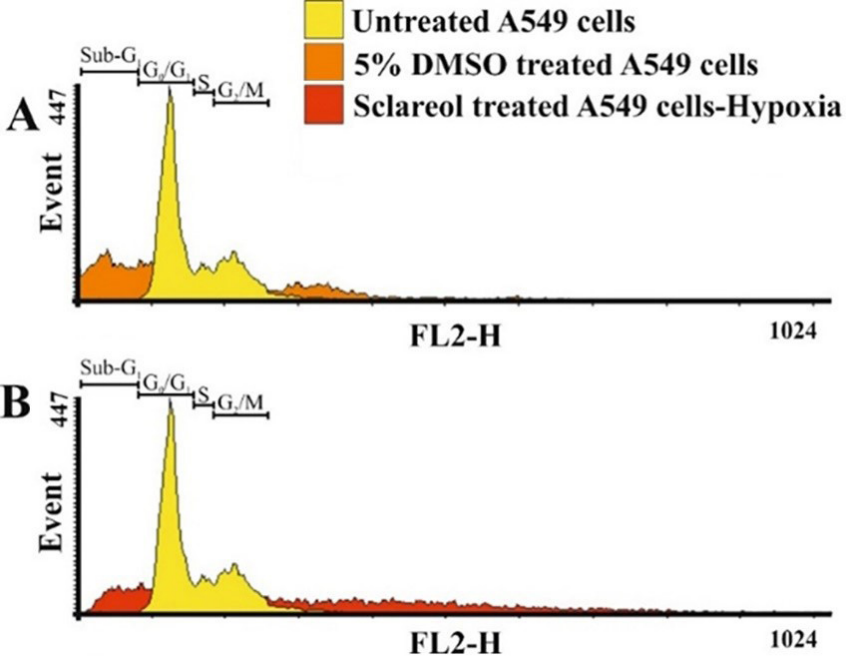
The distribution of cells in different phases of the cell cycle. The sub-G1 phase displays the apoptotic population of the treated cells.

PI staining was used to study the cell cycle of the sclareol-treated cells through flow cytometry analysis. The cell cycle assessment distinguishes cells in different phases of the cell cycle. DNA damages regulate the function of specific proteins involved in cell cycle checkpoints. The accumulation of cells in the sub-G1 phase of the cell cycle shows the cell cycle arrest and the occurrence of apoptosis. PI is routinely used for the staining of dsDNA which is highly accumulated in the nucleus in the G0/G1 and G2/M stages.^25,45^ As Figure 6shows, the untreated cells show a normal cell cycle distribution. But, the cell cycle of the most sclareol-treated cells shifts to the sub-G1, because of the occurrence of apoptosis. Cell cycle arrest by sclareol in hypoxia is another strong reason for the cytotoxicity of this compound and its ability to prompt apoptosis in cancer cells during hypoxia.

### Gene expression analysis

**Figure 7:**
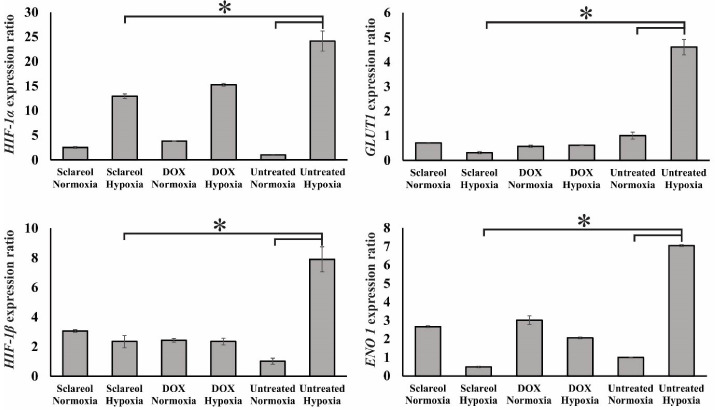
Gene expression analysis of *HIF-1α* and *HIF-1β*, *GluT1*, and *Eno1* in the untreated cells, sclareol/DOX treated- cells in hypoxia and normoxia. GAPDH was used as a housekeeping gene. The effect of sclareol on the expression of the genes in hypoxia was compared with the untreated negative control. Data represent the mean ± SD of three independent experiments. The asterisk represents significant differences between treatment conditions (*P* ≤ 0.05).

To study the effect of sclareol on the *HIF-1α*, *HIF-1β*, *GluT1*, and *Eno1* expression in A549 cancer cells in hypoxic and normoxic conditions, the qPCR technique was employed. The *GAPDH* gene was applied as a housekeeping gene. Figure 7 shows the expression level of the genes studied here. As it is clear, the *HIF-1α* and *HIF-1β* expressions are significantly higher in hypoxia than the normoxia in untreated cells (*P* < 0.05). Further, the expression of HIF-1 subunits in sclareol-treated cells was significantly reduced in hypoxia in contrast with the untreated cells (*P* < 0.05).

Moreover, in hypoxic cells, *GluT1* as a target gene for HIF-1 is significantly overexpressed compared to normoxic cells (*P* < 0.05) which leads to the metabolism changes to the glycolysis.^46^ As Figure 7 shows, the expression level of downstream *GluT1* and *Eno 1* genes were higher in untreated cells in hypoxia compared to normoxia (*P* < 0.05). However, sclareol downregulates their expression in hypoxia. Their expression in sclareol-treated cells is reduced synchronously with the reduction of HIF-1α expression level in hypoxia in comparison to the untreated cells. These results indicate the regulatory role of HIF-1 on the related downstream target genes in hypoxia and verify that cells reduce the harmful effect of oxygen deficiency by increasing the expression of glycolysis enzymes and excessive glucose uptake. Besides, the lowest expression of HIF-1 subunits was detected in untreated cells in normoxia in comparison to the other groups. Doxorubicin (DOX) (30 μM) was employed as a positive control. The expression level of HIF-1 subunits was reduced in DOX-treated cells in hypoxia. These findings indicate that the treatment of cells with DOX and sclareol in hypoxic conditions disrupts the compatibility of cells to hypoxia and reduces the expression of HIF-1 leading to cell death. The sclareol appears as a highly effective compound for inducing cell mortality in hypoxic conditions by downregulating target genes expression and adaptation of cells to hypoxia. However, additional tests are required to prove this point.

### Sclareol inhibits HIF-1 accumulation

The stability of the HIF-1α in the sclareol-treated cells was examined using western blot analysis in hypoxic and normoxic conditions.^47^ Besides, the β-actin protein was selected as an internal control. The alpha subunit of HIF-1 is precisely regulated by the amount of oxygen in the cells and acts as an oxygen sensor in the cells. So that in the normoxia, the alpha subunit is hydroxylated in specific amino acids and as a result, detected and destructed by the ubiquitin-proteasome pathway. Therefore, the HIF-1 protein is not assembled as an active transcription factor. While in the lower level of oxygen, the degradation of HIF-1α does not occur and both alpha and beta subunits assemble in the nucleus to form the active HIF-1. Besides, other factors are affecting the accumulation of HIF-1 and the nuclear translocation of HIF-1α. It was found that zinc suppresses erythropoietin production in hypoxic cells. It induces the accumulation and nuclear translocation of HIF-1α but inhibited the nuclear translocation of HIF-1β, leading to HIF-1 inactivation.^48^ Also, it was demonstrated that deacetylation of the cytosolic molecular chaperone Hsp70 by histone deacetylase (HDAC5) promotes HIF-1α interaction with Hsp90 and facilitates the rapid nuclear accumulation of HIF-1α. Also, the AMPK-mediated cytosolic translocation of HDAC5 is an active cellular response to hypoxia and low glucose stresses, which facilitates HIF-1 activation.^49^ In contrast, the active export of HIF-1 α from the nucleus was found to be important in the regulation of HIF-1 activity. Phosphorylation of the serine residues, close to an atypical nuclear export signal of HIF-1a by MAPK has been reported to block export by chromosome region maintenance 1 protein homologue (CRM1), thereby promoting nuclear accumulation and transcriptional activity.^50^ Further, it was found that thrombopoietin (TPO), an important and essential cytokine, is required for the normal development of hematopoietic stem cells. It induces HIF-1α expression in hematopoietic stem cells by enhancing its stability under normoxic conditions through the induction of mitochondrial reactive oxygen species (ROS). Elevated ROS changes the mitochondrial redox status, inactivates prolyl hydroxylase domain proteins (PHDs), and stabilizes the HIF-1α. Inhibition of mitochondrial electron transfer and use of ROS scavengers completely suppresses HIF-1α induction by TPO. Also, glucose metabolism is involved in the TPO-mediated HIF-1α induction. Inhibition of glucose transporter or glycolytic enzyme inhibited HIF-1α elevation. These results indicate that TPO induces HIF-1α expression similarly with hypoxia.^51^

**Figure 8:**
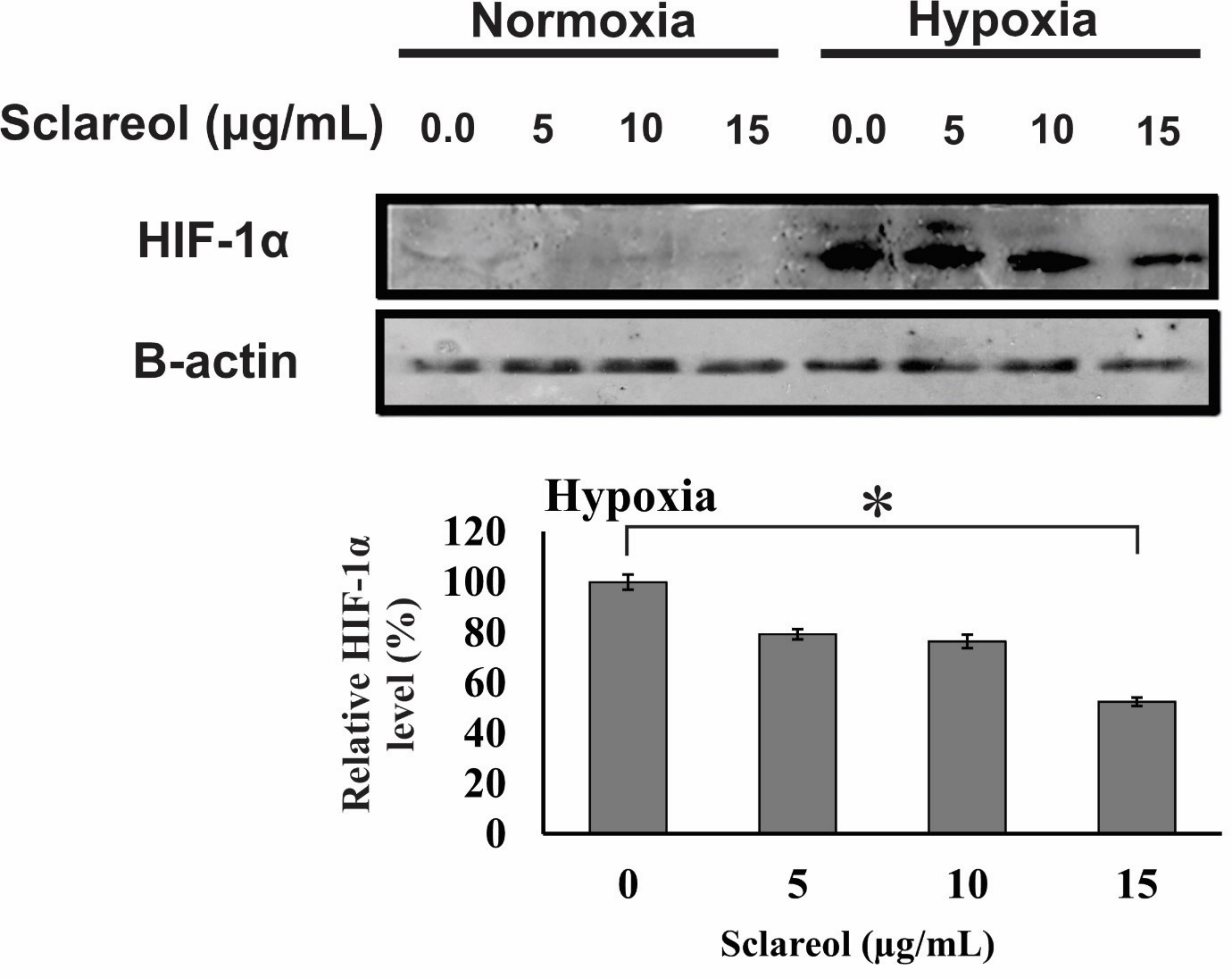
Western blot analysis of the alpha subunit protein of the HIF-1. A dose-response effect of sclareol is seen on the level of HIF-1α in treated cells in the hypoxic condition. The effect of sclareol on the accumulation of HIF-1α protein in hypoxia was compared with the untreated negative control. Data represent the mean ± SD of three independent experiments. The asterisk represents significant differences between treatment conditions (*P* ≤ 0.05).

HIF-1 activates some pathways which play a critical role in survival, cell proliferation, metabolism change, enhance of glucose uptake, and angiogenesis. As Figure 8 shows, the HIF-1α protein expression is extremely decreased in normal oxygen conditions. However, in hypoxia, the increased level of HIF-1α causes a higher level of HIF-1 protein. Our results showed that the expression of the alpha subunit was reduced in the sclareol-treated cells in a dose-related way in comparison to the untreated cells. Besides, sclareol reduced the alpha subunit expression level of HIF-1 at a concentration of 15 µg/mL significantly (*P* < 0.05). The results clearly show the effect of sclareol in reducing HIF-1α protein level and inhibiting the accumulation in hypoxic conditions.

## Conclusion

Sclareol, a labdane diterpenoid, is a natural compound found in the genus *Salvia* that is used in industry as a flavor. Various studies approved the cytotoxic effects of sclareol against different tumor cells. It has been shown that low oxygen level induces chemo-/radioresistance and tumor recurrence, and therefore reduces the therapeutic efficiency. In this research, the effects of sclareol on the A549 cells in hypoxia were assessed. Various techniques (e.g., MTT assay, genotoxicity assays, Annexin V/PI flow cytometry, qRT-PCR, western blot, and cell cycle analysis) verified the hopeful cyto/genotoxicity effects of sclareol on A549 cells in hypoxic condition. The obtained results showed decreased cell viability in sclareol-treated cells in a dose and time-dependent manner. Also, the results confirmed the geno-/cytotoxic properties of sclareol on A549 cells in hypoxia. Moreover, the regulatory role of HIF-1 on the downstream target genes (*Eno1*/*GluT1)* and downregulation of glycolysis enzymes and glucose uptake were demonstrated in sclareol-treated cells in hypoxia. Importantly, it was found that sclareol inhibits the accumulation of HIF-1 and downregulates downstream target genes, and induces cell death in hypoxic conditions by decreasing the adaptation of cells to hypoxia. In conclusion, the obtained findings showed the potential of sclareol, as a natural anti-tumor compound, in the inhibition of HIF-1 accumulation and disruption of the adaptation of epithelial lung cancer cells to hypoxia.

## Acknowledgments

The authors would like to express thanks to the Research Center for Pharmaceutical Nanotechnology (RCPN) for their technical support. This research was supported by Iran National Science Foundation (INSF) [grant number 93052212].

## Ethical Issues

None.

## Conflict of Interest

The authors state that there is no conflict of interest.
